# Broad-host-range vector system for synthetic biology and biotechnology in cyanobacteria

**DOI:** 10.1093/nar/gku673

**Published:** 2014-07-29

**Authors:** Arnaud Taton, Federico Unglaub, Nicole E. Wright, Wei Yue Zeng, Javier Paz-Yepes, Bianca Brahamsha, Brian Palenik, Todd C. Peterson, Farzad Haerizadeh, Susan S. Golden, James W. Golden

**Affiliations:** 1 Division of Biological Sciences, University of California San Diego, 9500 Gilman Dr., La Jolla, CA 92093, USA; 2 Scripps Institution of Oceanography, University of California San Diego, 9500 Gilman Dr., La Jolla, CA 92093, USA; 3 Institut de Biologie de I'Ecole Normale Supérieure, CNRS, UMR 8197, 46 rue d'Ulm, 75230 Paris, France; 4 Synthetic Biology Division, Life Technologies Corporation, 5791 Van Allen Way, Carlsbad, CA 92008, USA

## Abstract

Inspired by the developments of synthetic biology and the need for improved genetic tools to exploit cyanobacteria for the production of renewable bioproducts, we developed a versatile platform for the construction of broad-host-range vector systems. This platform includes the following features: (i) an efficient assembly strategy in which modules released from 3 to 4 donor plasmids or produced by polymerase chain reaction are assembled by isothermal assembly guided by short GC-rich overlap sequences. (ii) A growing library of molecular devices categorized in three major groups: (a) replication and chromosomal integration; (b) antibiotic resistance; (c) functional modules. These modules can be assembled in different combinations to construct a variety of autonomously replicating plasmids and suicide plasmids for gene knockout and knockin. (iii) A web service, the CYANO-VECTOR assembly portal, which was built to organize the various modules, facilitate the *in silico* construction of plasmids, and encourage the use of this system. This work also resulted in the construction of an improved broad-host-range replicon derived from RSF1010, which replicates in several phylogenetically distinct strains including a new experimental model strain *Synechocystis* sp. WHSyn, and the characterization of nine antibiotic cassettes, four reporter genes, four promoters, and a ribozyme-based insulator in several diverse cyanobacterial strains.

## INTRODUCTION

Cyanobacteria are a promising platform for production of renewable chemicals and fuels. Exogenous DNA can be introduced into cyanobacteria by transformation, conjugation, or electroporation, and can be propagated in a strain if carried on a replicating plasmid, or if integrated into the host chromosome. Extensive genetic tools have been developed for a select group of cyanobacterial strains, including autonomously replicating vectors, integration sites, selection markers, reporter genes and promoters (1,2). These molecular tools were originally developed to study fundamental cellular processes, but there is now an increasing interest in using cyanobacteria as cell factories for the production of biomolecules. Several strains have already been genetically engineered to produce fuel molecules, including ethanol, isobutyraldehyde, isobutanol, 2,3-butanediol and free fatty acids (3–5). These experiments were carried out in a few genetically manipulable model strains, primarily *Synechococcus elongatus* PCC7942 and *Synechocystis* sp. PCC6803. However, industrial-scale production of such molecules is likely to require the use of strains with greater potential for large-scale growth in outdoor ponds. Depending on the growth conditions and the products to be made, different production strains and compatible well-suited advanced genetic tools will be needed.

In comparison to *Escherichia coli* or *Bacillus subtilis*, engineering cyanobacterial strains requires special considerations because of their oligoploidy or polyploidy (6), the presence of restriction/modification systems (7) and their circadian rhythms (8). The cyanobacterial phylum is diverse and even the current model organisms differ from one another by their morphology, ecology, physiology and genomic content. Depending on these variations and their ability to undergo natural transformation, different protocols and culture conditions need to be applied for different strains. Genome size, genome content and codon usage can also be strikingly different from one strain to another. For example, the G+C content of the genome for *S*. *elongatus* PCC7942 is 55.4% whereas it is only 41.3% for *Anabaena* sp. PCC7120. Such variations may affect the ability of a particular strain to properly express an introduced gene of interest.

In order for cyanobacteria to be developed as useful biotechnological platforms, the process of engineering them must become streamlined. An underlying goal of synthetic biology is to make this engineering process easier (9) by defining and developing standards and by the characterization of genetic parts and devices. In the synthetic biology lexicon, a DNA fragment that performs a defined function is often referred to as a part. Multiple parts associated together to provide a higher order function are called a device, which is available as a specific module, and several devices linked with each other can be used to create a circuit. Synthetic biology is most advanced in just a few model organisms such as *E. coli, B. subtilis*, and *Saccharomyces cerevisiae*. It is much less advanced in cyanobacteria. A few genetic parts (promoters, reporters, terminators, etc.) and devices (antibiotic cassettes, broad-host-range replicons, etc.) are known to work in a few cyanobacterial strains but many parts and devices are strain-specific. Furthermore, those parts and devices that could qualify as broad host range are likely to vary in efficiency from strain to strain.

The BioBrick standard (10) has led to a large collection of parts in its Registry of Standard Biological Parts, including: promoters, ribosome-binding sites (RBSs), protein domains, protein coding sequences, translational units, terminators and plasmid backbones. Most parts and devices in the Registry operate in *E. coli* or *B. subtilis* and the Registry offers a limited number of backbones containing an origin of replication and an antibiotic resistance marker. Shetty *et al.* (9) developed an elegant and flexible system where a new vector can be assembled by combining replicon(s) and antibiotic resistance cassettes as standard modular parts. A number of schemes were built upon the BioBrick standard, and other standards (e.g. BglBrick, Golden Gate) were also designed based on traditional cloning and new assembly strategies (11–17). One synthetic biology approach dedicated to cyanobacteria was the construction of a BioBrick-compatible shuttle vector derived from the broad-host-range plasmid RSF1010, and the design and characterization of compatible biological parts including promoters, reporter genes and protein-degradation tags (18).

Here, we present an integrated and versatile platform for the construction of modular plasmids that can function in a broad range of cyanobacterial hosts. The modular plasmids include both autonomously replicating plasmids and suicide plasmids for gene knockout and knockin. Overall, the goals of this study were to (i) provide a new and efficient strategy (including an *in silico* design portal) for the construction of broad-host-range vector systems for cyanobacteria; (ii) develop standardized devices including new or improved parts essential for the construction of cyanobacterial host-vector systems; and (iii) characterize a number of these devices in diverse cyanobacterial strains.

## MATERIALS AND METHODS

### Strains and growth conditions

*E. coli* and cyanobacterial strains used in this study are listed in Supplementary Table S1. *E. coli* strains were grown at 37ºC with LB medium in tubes placed on a roller drum, or on agar plates, supplemented with appropriate antibiotics. Cyanobacterial strains *Anabaena* sp. PCC7120 (*A*. PCC7120), *Leptolyngbya* sp. BL0902 (*L.* BL0902), *Nostoc punctiforme* ATCC29133 (*N.* ATCC29133), and *Synechocystis* sp. PCC6803 (*S*. PCC6803) were grown in BG11 medium (19) as 50-ml or 100-ml cultures at 30ºC with continuous shaking or on agar plates (40 ml, 1.5% agarose) and continuous illumination of 70-μmol photons m^−2^ s^−1^. *Synechococcus elongatus* PCC7942 (*S.* PCC7942) was grown in similar conditions but with continuous illumination of 150-μmol photons m^−2^ s^−1^. Marine *Synechococcus* strains CC9311 (*S.* CC9311) and CC9605 (*S.* CC9605) were grown in SN medium (20) made with seawater obtained from the Scripps Pier (Scripps Institution of Oceanography, La Jolla, CA). *S.* CC9311 and *S.* CC9605 were incubated at 25°C with a constant illumination of 15- to 20-μmol photons m^−2^ s^−1^ and were maintained as 50-ml cultures in 125-ml glass flasks without shaking. We also evaluated our system in a recently isolated cyanobacterial strain, *Synechocystis* sp. WHSyn (*S.* WHSyn). *S.* WHSyn was isolated in 2001 from a Massachusetts salt marsh. Its 16S rRNA gene sequence is 99% identical to *S*. PCC6803. It can be grown in outdoor mini-ponds and in media ranging in salinity from freshwater (BG11) to seawater (data not shown). A more detailed report on the growth and other characteristics of *S.* WHSyn is in preparation (B.P.). In our experiments, *S.* WHSyn was grown in BG11 in conditions similar to those for *S*. PCC7942. The absence of *E. coli* in cultures of transconjugant cyanobacterial strains was tested by a lack of colony formation when samples were inoculated on LB plates incubated at 37ºC, and on BG11 Omni medium plates (BG11 supplemented with 0.04% (wt/vol) glucose and 5% (vol/vol) LB broth) incubated in the dark at 30ºC or on SN medium plates, supplemented with 0.2% (wt/vol) glucose and 0.05% (wt/vol) tryptone, incubated in the dark at 25ºC.

### Transformation of cyanobacteria

DNA was used to transform *S.* PCC7942 and integrated into the chromosome by homologous recombination using standard protocols that exploit its natural competence (21–23). Transformations of *A*. PCC7120, *L.* BL0902, *S*. PCC6803, *N.* ATCC29133 and *S*. WHSyn through biparental and triparental conjugations followed published protocols (24) modified from earlier methods (7,25). DNA was introduced into *S.* CC9311 and *S.* CC9605 via biparental conjugation following published protocols (26) modified from earlier methods (27).

### Construction of devices in donor plasmids

To construct donor plasmids, parts and devices were obtained by polymerase chain reaction (PCR) amplification from appropriate templates as one fragment or several overlapping fragments. In order to construct devices carrying adaptor sequences compatible with the assembly strategy described below, PCR primers contained adaptor sequences at their 5′-ends (Table 1). Primers were 5′-phosphorylated and the PCR products were then cloned into the *Eco*ICRI site of pUC19 following standard molecular biology techniques, or alternatively the PCR products were assembled using Gibson isothermal assembly (28) or a GeneArt Seamless Cloning and Assembly Kit (Life Technologies) with the plasmid backbone or only the ampicillin resistance cassette (when an *E. coli* origin of replication was a part of the device) obtained by PCR from pMA-RQ (Life Technologies). Devices harboring codon-optimized parts were constructed by Life Technologies. These devices were synthesized with the flanking sequences listed in Table 1 and a plasmid backbone carrying an ampicillin resistance marker. PCR amplifications were carried out with Phusion High-Fidelity DNA polymerase (Finnzymes, Thermo Scientific) according to the manufacturer's instructions. Information about the templates used to make parts and devices is provided in Supplementary Spreadsheet S1, Parts and Devices, through the CYANO-VECTOR portal (http://golden.ucsd.edu/CyanoVECTOR/), or in the GenBank files of the donor plasmids. The sequences of the donor plasmids were deposited in GenBank under the following accession numbers: KM017870 - KM017942.

**Table 1.** Summary of devices organized according to the GC-adaptors

Device category^a^	No. of devices^b^	R.E.^c^	GC-adaptors	Adaptor sequences^d^
Cyanobacterial replicons	11, 3	*Zra*I	G5C5-*Xba*I	5′-**GACGTC**GGGGGCCCCCGGGGGGATtctaga-3′
*E. coli* origin for knockout plasmids	2, 2		*Sac*I-C3G3	5′-**GACGTC**GGGCCCGGGCCCGGGGATgagctc-3′
*E. coli* origin to be assembled with a cyanobacterial replicon	2, 2	*Eco*RV	C3G3-*Mfe*I	5′-**GATATC**CCCGGGCCCGGGCCCGATcaattg-3′
			*Nhe*I-GC	5′-**GATATC**CGCGCGCGCGCGCGCGATgctagc-3′
Broad-host-range replicon	3, 3	*Zra*I	G5C5-*Xba*I	5′-**GACGTC**GGGGGCCCCCGGGGGGATtctaga-3′
Neutral sites	10,8		*Nhe*I-GC	5′-**GATATC**CGCGCGCGCGCGCGCGATgctagc-3′
Antibiotic resistance markers	17, 17	*Eco*RV	GC-*Nhe*I	5′-**GATATC**GCGCGCGCGCGCGCGGATgctagc-3′
			*Age*I-C2G	5′-**GATATC**CGGCGGCGGCGGCGGGATaccggt-3′
Cloning cassettes	1, 1	*Eco*RV	C2G-*Age*I	5′-**GATATC**CCGCCGCCGCCGCCGGATaccggt-3′
Expression cassette	2, 0		*Xba*I-G5C5	5′-**GATATC**CCCCCGGGGGCCCCCGATtctaga-3′
Reporter cassette	5, 2			
Promoter-reporter module	17, 15			

^a^A list of devices for each category is provided in the expanded Supplementary Table S2.

^b^The numbers of devices are presented as follows: no. of constructed, no. of tested.

^c^Restriction enzyme to be used for releasing the device from the donor plasmid.

^d^The *Eco*RV or *Zra*I restriction site to release the device from the donor plasmid is written in bold and uppercase, the core of the adaptor sequence is written in uppercase and an additional restriction site is written in lowercase.

### Construction of shuttle plasmids from selected devices

We define here as ‘shuttle plasmid’ any plasmid that can be propagated in both *E. coli* and a cyanobacterial host whether the plasmid autonomously replicates in the host or integrates into the host chromosome. Plasmid preparations were carried out with the QIAprep Spin Miniprep Kit (Qiagen). Restriction digests to release the devices from the donor plasmids were performed with the restriction enzymes *Zra*I or *Eco*RV-HF, according to the manufacturer's instructions (New England BioLabs). Restriction digests were typically performed for 3 to 5 h, using 5U of enzyme per μg of plasmid DNA in a final volume at least 50 times greater than the volume of enzyme added. DNA purification/concentration following restriction digests were performed with DNA Clean & Concentrator™-5 (Zymo). Nucleic acid concentrations were measured with a UV-Vis spectrophotometer NanoDrop 2000c. Assembly reactions were carried out using Gibson isothermal assembly (28) or a GeneArt Seamless Cloning and Assembly Kit (Life Technologies) following the authors’ or manufacturer's instructions with slight modifications described in the Results section.

### Antibiotic resistance assays

To determine the range of antibiotic concentrations in which selective growth may occur, three independent clones for each strain containing an antibiotic resistance gene and the wild-type strain were inoculated at OD_750_ of 0.1 in 2 ml of BG11 medium supplemented with the appropriate antibiotic. The experiments were carried out in 24-well plates at 30ºC under continuous illumination of 70–150 μmol photons m^−2^ s^−1^ for eight days. For some antibiotics, published data were used to establish antibiotic concentrations for strain construction experiments. For gentamicin and the codon optimized ampicillin and nourseothricin resistance markers, a range of antibiotic concentrations was used in mating experiments on plates and these data are noted in the Results section.

### Microscopy

Differential interference contrast (DIC) and fluorescence microscopy were carried out and images were captured on a Delta Vision (Applied Precision, Inc.) microscope system composed of an Olympus IX71 inverted microscope equipped with Olympus UPlanSApo 40x/0.90, 60x/1.35 and 100x/1.40 objectives and a CoolSNAP HQ2/ICX285 camera. Tetramethylrhodamine isothiocyanate (TRITC) filters (EX555/28 and EM617/73) were used to image autofluorescence of photosynthetic pigments, eCFP (EX436/10 and EM470/30), GFP (EX470/40 and EM525/36) and YFP (EX500/20 and EM535/30) filters were used to image heterologous protein fluorescence. Image acquisition, deconvolution and analysis were performed using Resolve3D softWoRx-Acquire (Version 4.0.0) and figure panels were constructed with Adobe Photoshop CS4.

### Fluorescence measurements

The emission intensities of fluorescent proteins from culture samples were measured in 96-well plates with a Tecan Infinite(R) M200 plate reader (TECAN). The excitation and emission wavelengths were set for eCFP to EX434/9 and EM477/20, for GFPmut2 and yemGFP to EX488/9 and EM518/20 and for YFP to EX490/9 and EM535/20. Measurements were taken from three independent clones for each strain grown for three days in liquid cultures. To obtain reproducible data, cultures were adjusted to an optical density at 750 nm of 0.2 at the start of growth, prior to induction (when needed), and finally prior to measurement.

### Gel image processing and statistical analysis

Gel band densities were determined with ImageJ 1.43 (http://rsb.info.nih.gov/ij/index.html). To assess statistical significance, the means were evaluated with one-way analysis of variance (ANOVA) followed by the Tukey's test (HSD) for multiple comparisons; Student's *t* test was used for pairwise comparisons. These analyses were performed with R 3.01 (http://www.r-project.org/).

## RESULTS

### Design of the assembly scheme

**Figure 1.**
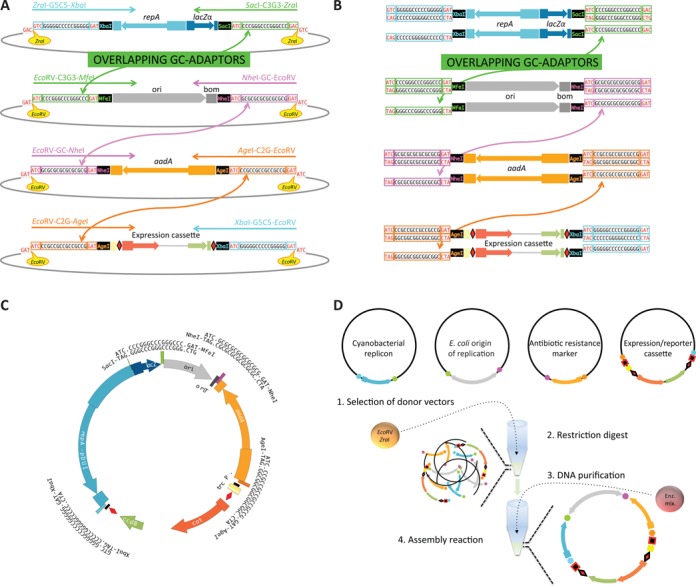
Strategy and method for combinatorial construction of plasmids, using a shuttle vector for *A*. PCC7120 as an example. (**A**) Four different donor plasmids harboring the devices required to build a destination vector for *A.* PCC7120 are shown. Each device is flanked by GC-adaptors that overlap with the GC-adaptors of the previous and following devices. The devices are released from the donor plasmid using the restriction enzyme *Eco*RV or *Zra*I. (**B**) During the assembly reaction, single-stranded overhangs are generated and anneal with the complementary sequences of adjacent fragments. The one-base-pair mismatch (G/T) in the overhangs that result from devices released from the donor plasmids using *Eco*RV and *Zra*I does not affect efficient assembly of the shuttle vectors. (**C**) Assembled shuttle vector for *A.* PCC7120 that contains four modules. (**D**) Overview of the steps required for the construction of a typical shuttle vector (see the text for details).

Our strategy for combinatorial construction of plasmids relies on short 21-nucleotide G- and C-rich overlapping sequences (GC-adaptors) and a standardized use of restriction sites. The overlapping sequences are used to guide the combination of the different components in a specific order and orientation (Figure 1). The GC-adaptors set the melting temperature of the overlapping sequences above 50ºC, the temperature at which the assembly reaction must be carried out with Gibson's isothermal assembly method (28).

Four GC-adaptors were devised: G5C5, C3G3, GC and C2G (Table 1). Cyanobacterial replicons or the *E. coli* origin of replication to be assembled into knockout plasmids are typically flanked by G5C5 and C3G3; *E. coli* origins of replication to be assembled with a cyanobacterial replicon are flanked with C3G3 and GC; broad-host-range origins of replication and homology regions for chromosome integration are flanked by G5C5 and GC; antibiotic resistance markers are flanked by GC and C2G and functional modules, including cloning/expression/reporter cassettes or promoter-reporter modules, are flanked by C2G and G5C5 adaptors.

Devices can be PCR amplified with primers carrying the GC-adaptors listed in Table 1 at the 5′-end of each primer or can be synthesized with the adaptor sequences. The devices are then subcloned in donor plasmids. The core sequences of the adaptors are 15-bp stretches containing G and C bases that are preceded with a recognition site for *Eco*RV or *Zra*I and followed by the first half of an *Eco*RV site, and finally another restriction site, typically *Xba*I, *Sac*I, *Mfe*I, *Nhe*I, or *Age*I.

During the first step of the construction of a new shuttle plasmid, the donor plasmids are digested with *Eco*RV or *Zra*I. *Zra*I was used instead of *Eco*RV when an *Eco*RV site was present within the device sequence. After the device is released from the donor plasmid, the second half of *Eco*RV or *Zra*I recognition sites (5′-ATC-3′ or 5′-GTC-3′) are present at both ends; the first half of the *Eco*RV site (5′-GAT-3′) after the G-C stretch insures complementarity between adaptors of the adjacent devices. When fragment ends generated with *Zra*I and *Eco*RV are joined, there is in a one-base-pair mismatch (G/T) within the adaptor sequence, but this does not prevent efficient assembly of the modules. The restriction sites that follow the overlapping sequences were added in case a GC-adaptor has to be removed or replaced, or if a device has to be replaced using traditional cloning. For this first step, the donor plasmids can be pooled together according to the restriction enzyme to be used; alternatively the digests can be done separately. Once the devices have been released from the donor plasmids, the digests are cleaned of enzymes, salts and other buffer components using a DNA clean-up/concentration kit and then assembled using Gibson isothermal assembly (28), GeneArt Seamless Cloning and Assembly (Life technologies), or similar technologies (16).

### Device selection for the construction of cyanobacterial shuttle plasmids

Over 70 devices were made following the scheme described above (Table 1 and Supplementary Spreadsheet S1, Devices). These devices can be assembled into a large variety of cyanobacterial plasmids that contain (i) the desired replicons or homology regions, (ii) an antibiotic-resistance marker and (iii) a functional device. The first category of devices contains either a dedicated origin of replication for *E. coli* or a broad-host-range origin that functions in both *E*. *coli* and cyanobacteria, and also typically contains an origin of conjugal transfer (*bom* site). If this device does not contain a broad-host-range origin, then it must be combined with a device that contains a dedicated cyanobacterial replicon or a homology region for chromosomal integration, with or without the counterselectable marker *sacB* for knockout and knockin plasmids. Antibiotic-resistance devices are self-explanatory. The functional devices include expression and reporter systems (Supplementary Spreadsheet S1, Devices). An automated annotation of each device was performed using a database of parts (Supplementary Spreadsheet S1, Parts) that was built based upon published sequences retrieved from GenBank. The automated annotation was carried out with scripts written using BioBIKE (29).

To construct a new shuttle plasmid, one or two devices, depending on the shuttle vector type, are chosen from each category. The construction of self-replicating cyanobacterial plasmids requires: an *E. coli* origin of replication, a cyanobacterial origin of replication, an antibiotic resistance marker and a functional module or custom sequence carrying the proper adaptors. The construction of broad-host-range vectors or neutral-site integration vectors follows the same construction scheme but because the broad-host-range replicon allows replication in many cyanobacteria and also in *E. coli*, it does not require a dedicated *E. coli* origin. Integration vectors containing neutral sites were constructed with an *E. coli* origin as part of the homology device and therefore do not require a separate *E. coli* origin part. The construction of gene knockout vectors requires a slightly different scheme. They require only two devices: an *E. coli* origin of replication (with or without *sacB)* designed to be assembled in knockout vectors and an antibiotic marker that is flanked by two custom PCR fragments that contain homology with the target sequences. In the next section, we describe devices by category or subcategory.

#### Cyanobacterial replicons

Traditionally, vectors for genetic manipulation of cyanobacteria have been chimerical molecules constructed from an *E. coli* plasmid, typically pBR322, and an endogenous cyanobacterial replicon isolated from the studied strain or a sister strain (30–35). We have cloned several of these cyanobacterial replicons (pDU1 for *A*. PCC7120; pFDA for *Fremyella diplosiphon* UTEX481; pDC1 for *N.* ATCC29133 and pANS for *S.* PCC7942) in donor plasmids compatible with the GC-adaptor assembly to build autonomously replicating shuttle plasmids that harbor the cyanobacterial replicon of choice (Table 1).

#### Improved broad-host-range RSF1010 replicon

**Figure 2.**
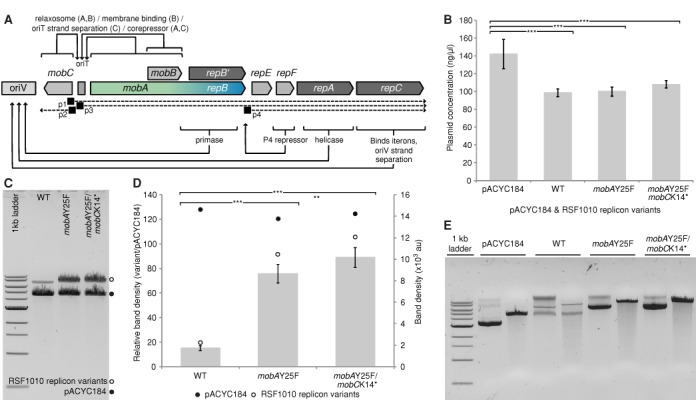
Site-directed mutation of the RSF1010 replicon improved plasmid yields. (**A**) Schematic representation of RSF1010 replicon with annotations (slightly modified from Meyer (37)). (**B**) Mean total DNA concentration ± SD of pACYC184 and RSF1010 WT replicon (pCVD046) and site-directed mutants from plasmid preparations carried out in triplicate on cultures normalized to the same amount of cells and grown from three independent colonies. (**C**) Electrophoresis of DNA on a 0.7% agarose gel from plasmid preparations of equal amounts of cells, determined by OD_600_, harboring pACYC184 and one of the RSF1010 variants linearized with *Eco*RV-HF. (**D**) Gel band densitometry of linearized RSF1010 variants compare to pACYC184. Bars represent the mean relative densities ± SD of RSF1010 variants over pACYC184, and open and close circles represent absolute averaged values for each plasmid. For each strain harboring pACYC184+RSF1010(WT), pACYC184+RSF1010(*mobA*Y25F), or pACYC184+RSF1010(*mobA*Y25F/*mobC*K14*), plasmid preparations were carried out on seven cultures normalized to the same amount of cells and grown from seven independent colonies. (**E**) Electrophoresis of DNA on a 0.7% agarose gel of pACYC184 and RSF1010 variants. Each lane contains 500 ng of undigested (left) plasmid DNA or plasmid linearized with *Eco*RV-HF (right). Statistical significances were inferred by the Tukey's test (HSD); ***P < 0.001, **P < 0.01, *P < 0.05, *.P* < 0.1.

RSF1010 is a self-mobilizable but not self-transmissible IncQ-group plasmid isolated from *E. coli* (36). The IncQ plasmids have been studied extensively (37) and are well known for their broad range of hosts, including a variety of cyanobacterial strains (24,27,38–41). However, RSF1010 manipulations often produce low yields of DNA after plasmid extraction, ineffective restriction digests and very low yield of DNA after purification for downstream applications. Furthermore, we found that isothermal assembly reactions were very inefficient. Its relatively low copy number (10 to 12 copies per cell in *E. coli* (42)) can in part explain these observations. However, we expected that these observations could also be due to its ability to self-mobilize. RSF1010 harbors an origin of transfer and encodes three proteins, which together make up the relaxosome. One of these proteins, MobA, reversibly nicks one of the DNA strands at the *oriT*, where it forms a stable complex covalently linked to the 5′-end of the cleaved/transferred strand. Nicked molecules denatured during the boiling or alkaline lysis step of plasmid preparations will result in single-stranded DNA useless for cloning. In addition, proteins bound to the plasmid DNA may also interfere with restriction digests or other enzymatic reactions. Several mutations are known to decrease the self-mobilization efficiency of RSF1010 (42,43), and an engineered plasmid lacking all known elements essential for mobilization has even been engineered (44). However, in addition to their role in mobilization, Mob proteins co-repress other RSF1010 genes and therefore are involved in control of plasmid copy number, stable maintenance and replication (37) (Figure 2A).

To reduce the self-mobility of RSF1010, while minimizing the impact on its gene regulation, we made two site-directed mutants of the RSF1010 replicon. Following the construction of a RSF1010 replicon as a device compatible with the GC-adaptor assembly (pCVD046), we made a conservative substitution in the active site of *mobA*. This mutation, *mobA*Y25F, is known to prevent cleavage at *oriT* and reduce the relative self-mobility of R1162, which is nearly identical to RSF1010, to 0.39% (43), while still allowing the relaxosome to form (45). Because MobC is known to co-repress expression of RSF1010 genes and a deletion of *mobC* was shown to increase copy number (42), we also made a second point mutation that introduced a stop codon early in the reading frame of *mobC*K14* to determine if this variant of the RSF1010 replicon would be further improved for plasmid constructions.

Whereas there was no significant difference in the concentrations of total plasmid DNA purified from equal numbers of cells carrying either the WT or mutant plasmids as measured by spectrophotometry (Figure 2B), gel electrophoresis of the same amounts of purified plasmid DNA showed different banding patterns between the pCVD046 (which contains the WT replicon of RSF1010) and the point mutants (Figure 2E). Plasmid pACYC184 was analyzed in parallel as a reference for plasmid conformations. While most of the purified DNA for pACYC184 and the point mutants of RSF1010-based plasmids were in a supercoiled conformation, only a small fraction of the WT RSF1010-based plasmid was isolated as supercoiled plasmid, leaving most of the DNA useless for downstream application, as demonstrated by incomplete restriction digestion of the WT plasmid (Figure 2E). To further quantify the relative amount of plasmid available for downstream applications, we co-transformed *E. coli* with a reference plasmid, pACYC184, and each of the three RSF1010 variants. Total plasmids were then isolated from triplicate cultures (using the same amount of cells), linearized with *Eco*RV, and run on an agarose gel (Figure 2C). Bands were quantified by densitometry and the relative amount of the RSF1010 variants and pACYC184 was calculated (Figure 2D). The results showed a 5- (*mobA*Y25F) to almost 6- (*mobA*Y25F/*mobC*K14*) fold increase in linearized plasmid for the point mutants compared to WT plasmid. The RSF1010(*mobA*Y25F) device was used in many of our subsequent experiments as our preferred broad-host-range origin of replication.

#### Neutral sites

Many cyanobacterial strains are able to undergo efficient homologous recombination. Sites on the chromosome where ectopic or heterologous sequences can be inserted with no effect on known phenotypes have been developed as knockin target sites for several strains of cyanobacteria including *S*. PCC6803 (46), *A*. PCC7120 (47–50), *S.* PCC7942 (23,51) and *Synechococcus* sp. PCC7002 (52). These target sites are often called ‘neutral sites’ (NS).

Three neutral sites have been used in *S.* PCC7942, and we have engineered these sites as devices compatible with the GC-adaptor assembly. Our further improvements of these NS devices included the addition of a tetracycline cassette and the replacement of the original *bom* site of pBR322, which requires a helper plasmid for conjugal transfer, with a minimal RK2/RP4 *oriT* (53). Based on the work cited above, we have also engineered two NS devices for *A*. PCC7120. Because *A*. PCC7120 does not undergo apparent double crossovers at a high frequency as does *S.* PCC7942, a *sacB* gene was added to these two NS devices for counterselection of double recombinants on medium supplemented with sucrose (54). The difference in the frequency of apparent double crossover events is likely a consequence of gene transfer by natural transformation in *S.* PCC7942 versus by conjugation in *A*. PCC7120 (55).

#### Knockout vectors

The ability of cyanobacterial strains to undergo homologous recombination can be used to perform gene knockouts by insertion, deletion or replacement. Two devices, one including an origin of replication for *E. coli* and an origin of transfer, and the other including the same parts and the *sacB* gene for counterselection of double recombinants, were engineered to allow the rapid construction of knockout vectors with an antibiotic cassette of choice (Table 1).

#### Antibiotic resistance markers

Eight antibiotics are commonly used for the selection of transformed cyanobacterial strains: ampicillin (Ap), chloramphenicol (Cm), erythromycin (Em), gentamicin (Gm), kanamycin (Km), neomycin (Nm), spectinomycin (Sp) and streptomycin (Sm). For several of these antibiotics, different cassettes with different promoters or genes have been used (1). We have constructed devices for each of the antibiotics listed above, including versions of the Ap and Cm resistance cassettes that were codon optimized for both *S.* PCC7942 and *A*. PCC7120. In addition, we constructed two codon-optimized cassettes for nourseothricin (Nt). In total, 17 antibiotic resistance devices were constructed and 9 of these were characterized in our core set of 4 strains (Supplementary Spreadsheet S1, Devices). Characterization of these devices in different strains is described below.

#### Functional modules

To characterize different promoter and reporter genes in a set of diverse cyanobacterial strains, we built four devices that contain the *E. coli* synthetic consensus promoter P*con*II (56,57) driving expression of four different reporter genes: YFP, yemGFP, GFPmut2 and eCFP. The P*con*II promoter of the P*con*II-YFP and the P*con*II-GFPmut2 devices was replaced with P*psbAI*, P*isiA* and P*phoA*, native to *S.* PCC7942, to evaluate the strength, host range and inducibility of these promoters. P*psbAI* was investigated as a potential constitutive promoter driving high expression levels (58). The open reading frame of *psbAI* encodes the photosystem II reaction center protein D1. P*phoA* is derived from the *phoA* gene, which encodes an alkaline phosphatase induced by inorganic phosphorus deprivation (59), and P*isiA* is derived from the *isiA* gene, which encodes a chlorophyll-binding protein induced by iron deprivation (60). P*phoA* and P*isiA* were investigated as inducible promoters (described below). Based on similarity searches, both genes are widely distributed and well conserved in cyanobacteria.

Protein expression can be greatly affected by translation efficiency, in particular translation initiation (61). Therefore, we have used our system to determine the utility of a synthetic RBS optimized in its genetic context, with *S.* PCC7942 as the reference organism, using an RBS calculator (61), and the ribozyme-based insulator sequence RiboJ (62) on expression of eCFP and YFP reporters (described below). In total, 17 promoter-reporter modules were constructed (Supplementary Spreadsheet S1, Devices).

#### Construction of new functional modules

New modules compatible with our GC-adaptor assembly scheme can be constructed from the ground up by any appropriate cloning method. To create new custom modules based on existing modules, one can PCR-amplify the parts of interest such as a promoter or reporter gene along with the GC-adaptor sequences and the vector backbone from an existing donor plasmid, and replace or add new parts as desired using appropriate cloning methods. Providing that the new device does not contain *Eco*RV (or *Zra*I) restriction sites, it can then be assembled into plasmids using the GC-adaptor assembly.

We have also constructed a module that contains a replaceable cassette that can be added to plasmids using the GC-adaptor assembly to construct vector backbones that include desired replicons or homologous regions for integration into the chromosome and an antibiotic-resistance marker. The module is designed to simplify the addition of a desired DNA fragment into a pre-constructed vector. This cloning cassette harbors a *ccdB* toxic gene (63), lethal for most *E. coli* cell lines, flanked by *Swa*I sites, adaptor sequences and terminators. PCR-amplified DNA fragments with the required adaptor sequences can be cloned by isothermal assembly into the vector backbone opened with *Swa*I. The adaptor sequences for this cassette (5′-TAGTCGGCCAATAACCCAGGGATTT-3′ and 5′-CTCCTGCCGGGGAGCTCCTTCATTT-3′) were designed so that single-stranded DNA would not form secondary structures, and so that the melting temperatures were above 50ºC.

### *In silico* construction of shuttle plasmids using the CYANO-VECTOR assembly portal

To organize and annotate the various devices/modules, facilitate the *in silico* construction of shuttle vectors, and encourage the use of this system; we have created a web service, the CYANO-VECTOR assembly portal (http://golden.ucsd.edu/CyanoVECTOR/, Supplementary Figure S1). It allows a user to build new shuttle plasmids from the collection of existing devices and from custom sequences, and then display them as maps or sequences, or export them as GenBank files (Supplementary Figure S1). The software also generates specific protocols for the *in vitro* construction of desired shuttle plasmids. A detailed description of the software is provided as Supplementary Method S1.

### *In vitro* construction of shuttle plasmids

Forty-two shuttle plasmids were constructed to characterize different devices (Supplementary Tables S3 and S4; Supplementary Spreadsheet S1, Shuttles) in selected cyanobacterial strains as described in the following section. In addition, 24 shuttle vectors harboring a cloning site or a cloning site flanking a promoter or a reporter gene were constructed for generic experiments (Supplementary Spreadsheet S1, Shuttles). A number of these shuttle vectors, including broad-host-range vectors and strain-specific vectors harbor different antibiotic resistance markers and the cloning cassette that contains the *ccdB* toxic gene. The efficiency of assembling the correct plasmid construct in these reactions was very high. For *E*. *coli* colonies possessing the correct antibiotic resistances, the number of correct plasmid constructs over the total number of constructs analyzed by restriction digests (Supplementary Tables S3 and S4, column P.A.E., plasmid assembly efficiency) allowed us to estimate the efficiency of the GC-adaptor assembly to be above 95%. The construction of new shuttle plasmids using the CYANO-VECTOR assembly portal is described in Supplementary Method S2.

### *In vivo* evaluation of selected devices in diverse cyanobacterial strains

The GC-adaptor assembly platform was used to assemble a variety of autonomously replicating and integrating plasmids that were then transformed into several cyanobacterial strains to characterize devices and parts, including antibiotic resistance markers, reporter genes, promoters, RBSs, and the ribozyme-based insulator sequence RiboJ. Parts and devices were generally evaluated in a core set of four diverse cyanobacterial strains including: *A*. PCC7120, *L.* BL0902, *S*. PCC6803 and *S.* PCC7942 (Supplementary Table S1). Additionally, our modified broad-host-range replicon RSF1010(*mobA*Y25F) was tested in *N.* ATCC29133, *S*. WHSyn and the marine *Synechococcus* strains CC9311 and CC9605. RSF1010(*mobA*Y25F) was used as the plasmid backbone for all strains except *S.* PCC7942, for which we used neutral-site NS1 harboring a tetracycline resistance cassette, NS1TC (Supplementary Spreadsheet S1, Devices).

#### Evaluation of replicons and neutral sites

Ten strain-specific replicons and 10 neutral sites for at least 4 different strains of cyanobacteria were built as devices for the GC-adaptor assembly. Several of these devices were used in the context of specific projects, which are not discussed in the present work. Information concerning the validation of these devices in particular strains of cyanobacteria can nevertheless be found as a part of the device description (Supplementary Spreadsheet S1, Devices).

Our collection of replicons also includes derivatives of the broad-host-range plasmid RSF1010, including the WT replicon and two site-directed mutant replicons (*mobA*Y25F and *mobA*Y25F/*mobC*K14*) that were engineered to produce greater yields of useful plasmid DNA for downstream cloning applications (as described above). The three replicons were assembled with the *aadA* device, which confers resistance to spectinomycin and streptomycin, and the GFPmut2 reporter gene driven by the P*con*II promoter into shuttle plasmids that were conjugated into 3 strains: *A*. PCC7120, *L.* BL0902, and *S*. PCC6803. Both the WT and the *mobA*Y25F mutant were successfully conjugated into these three strains. The *mobA*Y25F/*mobC*K14* mutant was successfully conjugated into *A*. PCC7120 and *L.* BL0902 but at much lower efficiencies, and the conjugation did not succeed in *S*. PCC6803. To investigate how these mutations would affect the efficiency of transfer by conjugation, the three versions were conjugated into *A*. PCC7120, *L.* BL0902, and *S*. PCC6803 as follows. Biparental matings were carried out so that the same amount of cyanobacteria and *E. coli* cells were used for the three constructs, and serial dilutions of the mating mixtures were spotted on plates. Although exconjugant colonies are always obtained in routine experiments, transformation efficiencies of the *mobA*Y25F mutant were reduced by 2 to 4 orders of magnitude and we did not obtain any exconjugants with the *mobA*Y25F/*mobC*K14* mutant (Supplementary Figure S2). The *mobA*Y25F mutant was also further successfully conjugated into *N.* ATCC29133, *S*. WHSyn and the marine *Synechococcus* strains CC9311 and CC9605, suggesting that the host range of the replicon was not affected by the point mutation Y25F in *mobA*.

The replicative stability of RSF1010(*mobA*Y25F) was assessed by PCR carried out on cultures grown for 4 to 8 months in liquid media supplemented with the appropriate antibiotic. For *A*. PCC7120, *L.* BL0902*, S*. PCC6803 and *S.* WHSyn, overlapping PCR products covering the entire plasmid were obtained using three sets of primers (Supplementary Figure S3). In agreement with the fact that fluorescent protein reporters encoded on the plasmid were still detectable by microscopy, these data show that the plasmid was stably maintained in these strains with antibiotic selection. However, PCRs detected the plasmid in *E. coli*-free *S.* CC9311 and *S.* CC9605 cultures that had been transferred three times in liquid over a period of one and a half months, but not in those transferred eight times in liquid over a period of four months (Supplementary Figure S3). These results suggest that the plasmid was lost over time for these two strains, presumably following the acquisition of chromosomal mutations conferring resistance to kanamycin. In our experience, *S.* CC9311 and *S.* CC9605 can develop spontaneous resistance to kanamycin (data not shown).

#### Evaluation of antibiotic resistance markers

**Figure 3.**
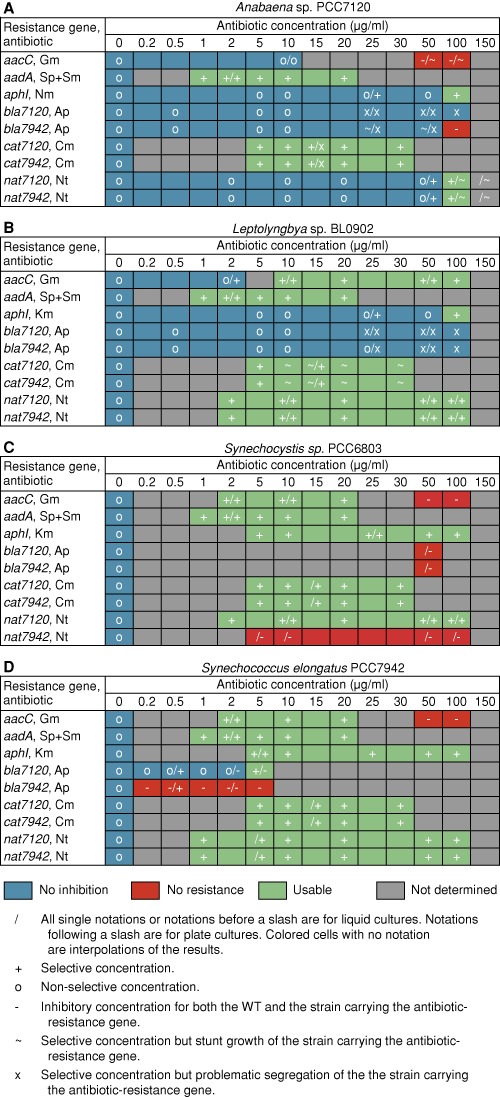
Characterization of antibiotic-resistance genes. The growth of the four cyanobacterial strains *A*. PCC7120, *L*. BL0902, *S*. PCC6803, and *S*. PCC7942 (**A**)–(**D**) harboring antibiotic resistance genes for gentamicin (Gm), spectinomycin + streptomycin (Sp+Sm), kanamycin or neomycin (Km or Nm), ampicillin (Ap), chloramphenicol (Cm) and nourseothricin (Nt) were determined for different concentrations of the corresponding antibiotic. Data are from triplicate cultures of three independent clones for each strain. Data are from liquid cultures unless a forward slash is shown, in which case the data are from liquid cultures (left of slash) or agar plate cultures (right of slash). Colored cells without notations are interpolations of flanking data. Selective concentrations are those that inhibit growth of the WT and allow growth of strains carrying the antibiotic-resistance gene.

To evaluate the nine antibiotic resistance markers, 18 shuttle plasmids were constructed and introduced into selected cyanobacteria (Supplementary Table S3). To provide a second marker that allows screening for plasmid transfer and maintenance, the shuttle plasmids also carried a fluorescent reporter device, either YFP (7942NS1TC) or GFPmut2 (RSF1010(*mobA*Y25F)), driven by P*con*II. The ranges of effective antibiotic concentration for the nine antibiotic markers are summarized in Figure 3. The *aadA* cassette conferred a broad-host-range resistance to both Sp and Sm. In addition, it offers a large window of resistance in all strains tested. Each WT strain was killed by the lowest concentration of Sp and Sm tested, whereas strains harboring the antibiotic marker grew at concentrations of these antibiotics that were more than one order of magnitude higher. Similarly, the two versions of the *cat* gene, *cat7120* and *cat7942*, functioned in all four strains and provided resistance across a useful range of chloramphenicol concentrations. However, on *A.* PCC7120 mating plates, we observed a significant background growth of WT on chloramphenicol. The *aphI* cassette confers resistance to a wide range of Km concentrations in *S.* PCC7942 and *S*. PCC6803. The range of concentrations is limited for *L.* BL0902, and Km must be replaced by Nm in *A*. PCC7120, because it is intrinsically resistant to Km. The *aacC1*, *nat7120*, and *nat7942* antibiotic resistance markers conferred a variable spectrum of usability depending on the host strain (Figure 3).

Versions of the antibiotic-resistance genes *bla*, *cat* and *nat* were codon optimized for *S.* PCC7942 or *A*. PCC7120, which have different G+C content and codon usage. The only notable difference for these codon-optimized antibiotic-resistance genes was that *nat7942* did not work in *S*. PCC6803. For the two ampicillin-resistance genes, *bla7942* and *bla7120*, we were unable to obtain fully segregated clones in *A*. PCC7120, *L.* BL0902, or *S*. PCC6803 even after several generations of growth and multiple restreaks onto fresh solid medium. In *S.* PCC7942, unexpectedly, *bla7120* provided usable resistance at 5 μg/ml but *bla7942* did not work well for unknown reasons.

#### Evaluation of reporter and expression systems

**Figure 4.**
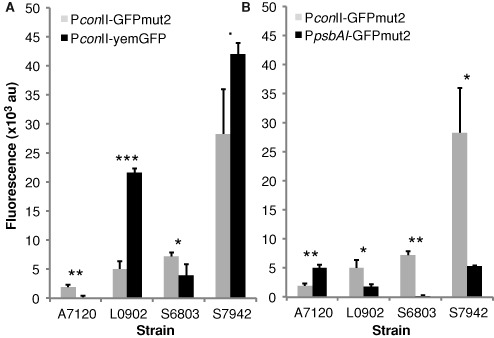
Fluorescence intensities of three promoter-reporter modules in four cyanobacterial strains. (**A**) Comparison of two GFP variants, GFPmut2 and yemGFP driven by P*con*II. (**B**) Comparison of the promoter activities of P*con*II and P*psbAI*(S7942) driving GFPmut2. The data are the mean ± SD of triplicate cultures grown from three independent colonies adjusted to OD_750_ of 0.2 (0.1 for A7120). Statistical significances were inferred by the Student's *t* test; ***P < 0.001, **P < 0.01, *P < 0.05, *.P* < 0.1. Strain labels are: *Anabaena* sp. PCC7120 (A7120), *Leptolyngbya* sp. BL0902 (L0902), *Synechocystis* sp. PCC6803 (S6803) and *Synechococcus elongatus* PCC7942 (S7942).

Twenty-four shuttle plasmids (including two control plasmids) were constructed and introduced into the core set of four strains to characterize reporter and expression parts (Supplementary Table S4). The expression of four fluorescent proteins, eCFP, GFPmut2, yemGFP and YFP, driven by the P*con*II promoter were characterized. Levels of fluorescence obtained for eCFP and YFP were not detectable or too low to be properly quantified. Improvements of these reporter systems were carried out and are described below. Levels of fluorescence for GFPmut2 and yemGFP were suitable for quantification. As shown in Figure 4, fluorescence emission can vary significantly from strain to strain. The comparison of two derivatives of GFP, GFPmut2 and yemGFP, showed that GFPmut2 could be used in any of the studied strains (Figure 4A). Fluorescence intensities measured for yemGFP were significantly higher (4–5 fold) in *L.* BL0902 and notably higher in *S.* PCC7942 compared to GFPmut2, but yemGFP was not detectable in *A*. PCC7120. Fluorescence of GFPmut2 was significantly higher than yemGFP in *S*. PCC6803.

Four promoters were characterized in the core set of four strains, where they showed significantly different levels of reporter activity. The synthetic P*con*II promoter and the P*psbAI* promoter from *S*. PCC7942 were expected to be constitutively expressed and relatively strong under our growth conditions. The P*con*II promoter functioned in all four strains. Fluorescence levels obtained with P*psbAI* were significantly lower than those for P*conII* in all strains except *A*. PCC7120, and P*psbAI* did not produce detectable fluorescence in *S*. PCC6803 (Figure 4B).

**Figure 5.**
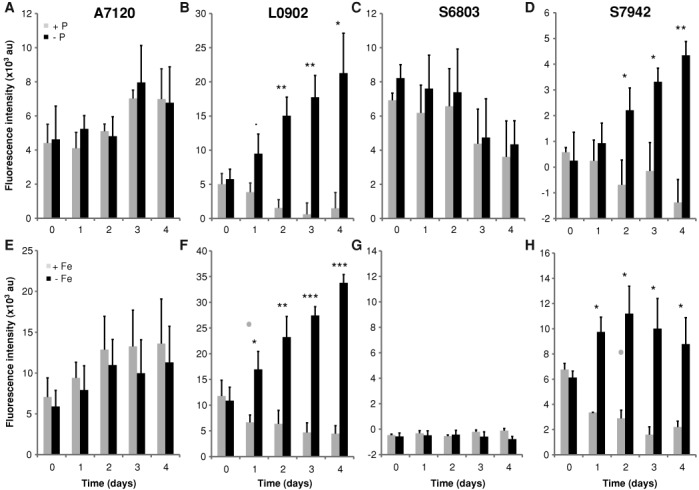
Promoter activities for P*phoA* and P*isiA* driving GFPmut2 in inducing and non-inducing conditions over a time course of four days post induction in four cyanobacterial strains. (**A**)–(**D**) P*phoA* activities in phosphate replete (gray bars) versus depleted (black bars) cultures. (**E**)–(**H**) P*isiA* activities in iron replete vs. depleted cultures. Promoter activities were measured as GFPmut2 fluorescence ± SD of triplicate cultures grown from three independent colonies adjusted to OD_750_ of 0.2 (0.1 for A7120). Statistical significances were inferred by the Student's *t* test; ***P < 0.001, **P < 0.01, *P < 0.05, *.P* < 0.1. Strain labels are as in Figure 4.

The regulated P*phoA* and P*isiA* promoters driving GFPmut2 were characterized over a period of five days following phosphorus or iron deprivation, respectively. Different responses across strains were observed (Figure 5). For strains carrying the *phoA* promoter, significant differences in fluorescence were observed in *S.* PCC7942 and *L.* BL0902 following phosphate deprivation with almost 10-fold higher expression by day 2 for the induced cultures of *L.* BL0902 (Figure 5A–D). Fold induction of induced over non-induced cultures could not be calculated for *S.* PCC7942 because fluorescence levels of the non-induced cultures were below the detection threshold. However, there was a 9-fold increase in fluorescence between the induced culture at day 2 compared to day 0. For *L.* BL0902, substantial fluorescence levels were observed in both induced and non-induced cultures at the start of the experiment; therefore, only a 2.6-fold increase of fluorescence intensity on day 2 compared to day 0. Fluorescence levels of the non-induced cultures decreased upon transfer of the strains to fresh medium. For *A*. PCC7120 and *S*. PCC6803, we observed substantial levels of fluorescence but no significant difference between induced and non-induced cultures.

The results for strains carrying the P*isiA* promoter followed the same trends that were found with P*phoA*. Significant increases in reporter fluorescence of 2.5- to 3-fold were observed in *S.* PCC7942 and *L.* BL0902 starting on the first day following iron deprivation (Figure 5E–H). Fluorescence intensities were about 6-fold higher in the induced strains by the third day following iron deprivation. For both of these strains, cultures showed significant levels of fluorescence on day 0, and fluorescence levels of non-induced cultures decreased after transfer to fresh medium. *A*. PCC7120 showed moderate levels of reporter fluorescence and no significant difference between induced and non-induced cultures. Reporter fluorescence levels for both induced and non-induced cultures of *S*. PCC6803 were below the detection threshold.

**Figure 6.**
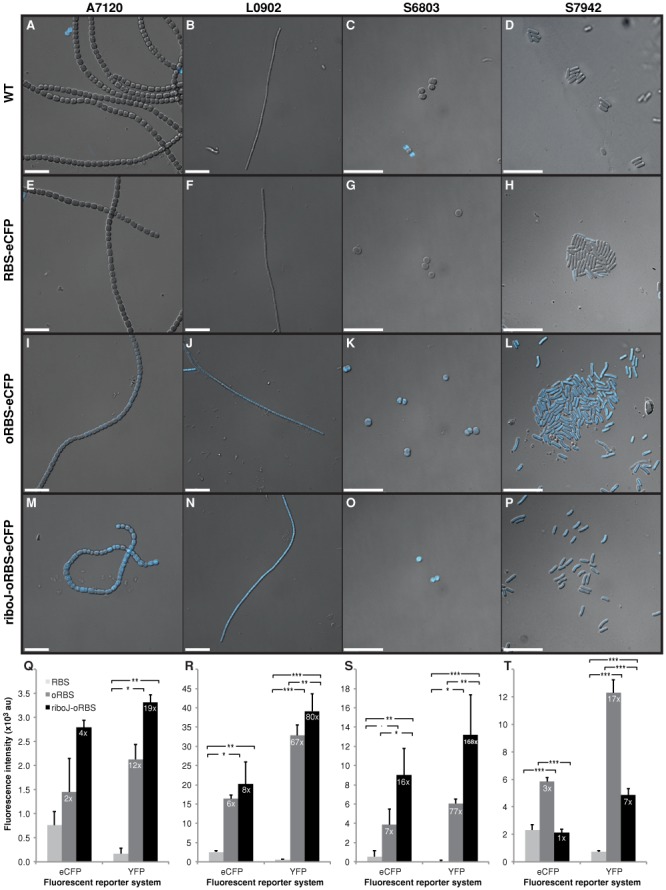
Levels of eCFP fluorescence produced by different ribosome-binding sites and a ribozyme-based insulator sequence in four cyanobacterial strains. (**A**)–(**P**) Photomicrographs of WT strains and strains harboring eCFP driven by P*con*II and a synthetic RBS, an optimized synthetic RBS (oRBS), or a RiboJ sequence upstream of the oRBS. Images are merged differential interference contrast (DIC) and cyan fluorescence photomicrographs. In the photomicrographs A and C, blue fluorescing cells represent dying WT cells that have lost autofluorescence. (**Q**)–(**T**) Mean eCFP and YFP fluorescence intensities ± SD of triplicate cultures grown from three independent colonies normalized to OD_750_ of 0.2 (0.1 for A7120). Fold changes of fluorescence intensities for the oRBS and the RiboJ-oRBS over the original synthetic RBS are shown. Statistical significances were inferred by the Tukey's test (HSD); ***P < 0.001, **P < 0.01, *P < 0.05, *.P* < 0.1. Strain labels are as in Figure 4.

We found that we could significantly improve the expression of eCFP and YFP reporters with an optimized RBS ([A/G]AAGGAGGT centered 12 nt upstream of the start codon) and the ribozyme-based insulator sequence RiboJ (62) (Figure 6). With the optimized RBS alone, YFP showed a significant and sometimes substantial increase in expression in all four strains *A*. PCC7120, *L.* BL0902, *S*. PCC6803 and *S.* PCC7942 (Figure 6Q–T). Addition of RiboJ upstream of the optimized RBS resulted in a further increase in YFP expression in three strains, but resulted in decreased expression in *S*. PCC7942 for unknown reasons. The results were similar for eCFP, except that the optimized RBS alone produced only a small non-significant increase in the reporter expression in *A*. PCC7120.

## DISCUSSION

In the past, genetic tools typically have been developed individually for specific cyanobacterial strains and for specific functions. In general, these tools have not been designed to be modular or to work in diverse strains, and there has been a lack of standardization and characterization of their component parts. Standard biological parts (http://partsregistry.org) and assembly schemes (e.g. BioBrick and BglBrick) have been designed for the construction of synthetic genetic systems. However, these systems are often centered on protein expression and operate in only a few widely used organisms such as *E. coli*, *B. subtilis*, or yeast. In contrast, the GC-adaptor assembly system described here is based on a modular vector backbone, including parts for plasmid replication in *E. coli*, conjugal transfer, antibiotic resistance and plasmid replication or chromosomal integration in cyanobacteria, and allows the construction of plasmids for a broad range of cyanobacterial hosts. The assembly scheme allows the construction of different types of vectors, including self-replicating broad-host-range vectors and vectors specific to particular cyanobacterial strains, as well as integration (suicide) vectors for genetic knockin and knockout. Because of the modularity of this system, multiple compatible self-replicating plasmids and integration plasmids can be constructed for a particular strain, which allows the production of heavily engineered strains that contain multiple genetic modifications. Many experimental designs require the use of multiple integration sites or autonomously replicating plasmids and multiple resistance markers. For example, the study of circadian rhythms in *S.* PCC7942 can require engineering a strain with a reporter device to follow rhythmicity, a gene knocked-out, a WT copy of the gene for testing complementation, and a gene tagged with a reporter system for determining protein localization (23). As another example, the production of isobutyraldehyde in *S.* PCC7942 was achieved by the heterologous expression of five different genes from three distinct integration sites in the chromosome including two neutral sites (64).

The construction of engineered cyanobacterial strains relies either on chromosomal integration through homologous recombination or on autonomously replicating plasmids. Based on previous studies (23,47–51), we have engineered several neutral sites as devices compatible with the GC-adaptor assembly for *S.* PCC7942 and *A*. PCC7120. Homologous recombination is also used to perform gene knockouts or gene modifications. Modules that contain an origin of replication for *E. coli*, an origin of transfer, and a *sacB* gene for counterselection were produced as devices to allow the construction of knockout plasmids.

Chimerical plasmids constructed from an *E. coli* plasmid and an endogenous cyanobacterial plasmid have been often used as cyanobacterial shuttle vectors (30–34,35). We constructed several cyanobacterial replicons as devices compatible with the GC-adaptor assembly for *A*. PCC7120, *F. diplosiphon* UTEX481, *N.* ATCC29133 and *S.* PCC7942. Some strain-specific cyanobacterial replicons may have a broader host range than currently known, because, for example pDU1 was isolated from *Nostoc* PCC7524 but is known to replicate in several phylogenetically distant strains, including *A*. PCC7120, *Anabaena* M-131 (65), *Fischerella muscicola* PCC7414, *Chlorogloeopsis fritschii* PCC 6912 (66), *Chroococcidiopsis* spp. str. 029, 057 and 123 (67) and *Oscillatoria* MKU 277 (68).

Alternatively, derivatives of the broad-host-range plasmid RSF1010 are known to replicate in numerous cyanobacterial strains (24,27,38–41). RSF1010 is, however, a poor cloning vector, which we suspected was due to its capability to self-mobilize. To circumvent this problem, we performed a conservative substitution in the active site of *mobA*, Y25F, known to prevent cleavage at *oriT* and almost completely suppress self-mobility of RSF1010 (43). This resulted in a variant of the plasmid, RSF1010(*mobA*Y25F), that produces 5-fold more usable DNA for cloning experiments, has better seamless cloning efficiencies, and that retained its broad-host-range properties. Although conjugation efficiency was reduced by 2 to 4 orders of magnitude, this was not a limitation in our experiments, and it is possible that a WT copy of *mobA* could be provided in trans on a helper plasmid to improve the conjugation efficiency for experiments that require it. A second point mutation that introduced a stop codon early in the reading frame of *mobC*, the product of which is known to co-repress the expression of RSF1010 replication genes (42), resulted in a plasmid that produced slightly more usable DNA but this variant had dramatically reduced conjugation efficiency for *A*. PCC7120 and *L.* BL0902, and conjugations failed for *S*. PCC6803. Our results showed that the RSF1010(*mobA*Y25F) plasmid is stable in most strains grown under antibiotic selection. However, after several months of selective growth, two marine *Synechococcus* strains lost the plasmid, presumably because they acquired spontaneous resistance to the selective antibiotic.

Cyanobacterial genetics is largely based on antibiotic selection of transformants carrying an antibiotic-resistance marker. Therefore, having a panel of antibiotic markers to choose from facilitates the construction of heavily engineered strains. Seventeen variations of antibiotic markers for six classes of antibiotics were constructed as devices compatible with the GC-adaptor assembly system and nine were characterized in selected cyanobacterial strains. Not surprisingly, we found that the antibiotic markers performed differently in different strains. However, some, such as the *aadA* gene, which provides selection with spectinomycin and streptomycin, displayed large windows of usability in a broad range of hosts. Other antibiotic-resistance markers were also found to be useful to various degrees in different strains, including the *cat* genes and the two newly characterized codon-optimized *nat* genes conferring nourseothricin (Nt) resistance.

In this work, we have characterized the expression of four fluorescent reporter proteins, eCFP, GFPmut2, yemGFP and YFP, driven by the P*con*II promoter in several strains. Reporter fluorescence was considerably different from strain to strain. The GFPmut2 worked well in all of the studied strains. In comparison to GFPmut2, fluorescence from yemGFP was significantly higher (4–5 fold) in *L.* BL0902 and notably higher in *S.* PCC7942 but significantly lower in *S.* PCC6803 and not detectable in *A*. PCC7120. eCFP and YFP reporters constructed using the same promoter and RBS as was used for GFPmut2 and yemGFP did not work in any of these four strains. As discussed below, the eCFP and YFP reporters were improved by incorporating optimized RBSs and RiboJ insulator sequences.

Promoters are key components in synthetic biology. Several promoters, including promoters responsive to environmental stimuli, have been characterized to understand fundamental biology processes and some of these have been applied to drive the expression of genes in native or heterologous pathways. Most promoters have been studied or used in their native organisms (1,69) but a few heterologous promoters, *con*II, T7, *tac*, *tet* and *trc*, have been used in cyanobacteria. Detailed characterization of the *trc* and *tet* promoters in *S*. PCC6803 has been carried out by Huang *et al.* (18) and Huang and Lindblad (70), respectively. We focused on characterizing two constitutive promoters, P*con*II and P*psbAI* and two inducible promoters, P*phoA* and P*isiA*. The P*psbAI* promoter is regulated by light (58), but is constitutively expressed under our growth conditions. Reporter fluorescence levels obtained with P*psbAI* were significantly lower than with P*con*II in all strains except *A*. PCC7120. For strains carrying the P*phoA* promoter or the P*isiA* promoter, different responses across strains were observed. Both promoters can be used in *L.* BL0902 and *S.* PCC7942 as they were mostly off in replete medium and they were induced in nutrient-depleted medium. However, these two promoters did not show regulation in *A.* PCC7120 or *S.* PCC6803, and P*isiA* was not functional in *S.* PCC6803.

For optimal gene expression, codon usage affects the rate of translation but in most cases translation initiation is the rate-limiting step. Initiation depends on the hybridization of the 16S rRNA to the RBS, the binding of the tRNA^fMet^ to the start codon, the spacing between RBS and the start codon and the presence of RNA secondary structures that occlude either the 16S rRNA binding site or the standby site (61). Typical methods for heterologous protein expression in cyanobacteria often rely on either the native RBS associated with the promoter being used or the RBS associated with the open reading frame to be expressed. In either case, using identical RBS sequences in different genetic contexts can result in different protein expression levels. To determine if translation initiation was responsible for the poor expression of eCFP and YFP, we tested constructs containing optimized RBS sequences, which were optimized in their genetic context using an RBS calculator (61), and the optimized RBS sequences headed by the ribozyme-based insulator sequence RiboJ (62). Overall, our results showed significantly improved expression of eCFP and YFP in all four tested strains, with the exception that RiboJ decreased the expression of eCFP and YFP in *S*. PCC7942 for unknown reasons.

Libraries of promoters, translation initiation elements and transcription terminators have been characterized in *E. coli* and correlated with models to reliably predict the quantitative behaviors of novel combinations of genetic parts (71,72). Genetic circuits including logic gates and devices that allow rewritable data storage have been engineered in *E. coli* (73,74). However, the field of synthetic biology for cyanobacteria is much less advanced and we cannot rely on predicted behaviors based on results in *E. coli* to apply to different cyanobacterial strains. In fact, we found large variations among cyanobacterial strains for the usability of antibiotic resistance markers, reporter genes, and promoters. Therefore, parts and devices need to be characterized and validated in each cyanobacterial strain. The task is not trivial, but is required to reach one goal of synthetic biology: the ability to engineer complex circuits based on predictive models.

This work provides an integrated and expandable platform for the efficient construction of vectors and finished plasmids from *in silico* design to laboratory protocols. An assembly strategy, many biological devices, bioinformatics tools and improved protocols were devised to provide the necessary tools to construct a wide range of plasmids for genetic experiments and genetic engineering of a broad range of diverse cyanobacteria. Forty-two shuttle plasmids were assembled and introduced into select cyanobacteria to characterize 23 modules or devices. In addition, we constructed 24 vectors harboring cloning sites, different antibiotic resistance markers and different neutral sites or the broad-host-range replicon variant of RSF1010 (Supplementary Spreadsheet S1, Shuttles). Fifty-three different devices have been tested so far.

The combinatorial construction of shuttle vectors was shown to be very efficient and this platform is easily expandable by addition of new biological devices carrying appropriate GC-adaptor overlapping sequences that are compatible with our system. In this work, devices and shuttle vectors were constructed for cyanobacteria but many of the devices as well as the construction scheme are applicable to other organisms. For example, RSF1010-based plasmids are known to replicate in a broad range of hosts other than cyanobacteria, and other replicons and integration devices can be added to the platform.

Up until a few years ago, cyanobacterial genetics and engineering have mostly been developed in a relatively small number of academic and research laboratories. However, over the past decade, cyanobacteria have become targeted organisms for synthetic biology and metabolic engineering by an increasing number of laboratories and companies that are developing cyanobacteria as photosynthetic production platforms. Our improved genetic toolbox for cyanobacteria, which includes a wide variety of parts and devices for genetic manipulations in a variety of cyanobacterial strains, will greatly benefit the research and industrial communities.

## SUPPLEMENTARY DATA

Supplementary Data are available at NAR Online.

SUPPLEMENTARY DATA
