# Supplementary material for: Broad-host-range vector system for synthetic biology and biotechnology in cyanobacteria
Source: Nucleic Acids Res. 2014 Jul 29;42(17):e136. doi: 10.1093/nar/gku673 (PMC4176158; doi:10.1093/nar/gku673)
Supplement: SUPPLEMENTARY DATA [file supp_42_17_e136__index.html]

Broad-host-range vector system for synthetic biology and biotechnology in cyanobacteria — Broad-host-range vector system for synthetic biology and biotechnology in cyanobacteria — SUPPLEMENTARY DATA 

# Broad-host-range vector system for synthetic biology and biotechnology in cyanobacteria

## SUPPLEMENTARY DATA

**Files in this Data Supplement:**

- SUPPLEMENTARY DATA
- SUPPLEMENTARY DATA
