# Supplementary material for: Broad-host-range vector system for synthetic biology and biotechnology in cyanobacteria
Source: Nucleic Acids Res. 2014 Jul 29;42(17):e136. doi: 10.1093/nar/gku673 (PMC4176158; doi:10.1093/nar/gku673)
Supplement: SUPPLEMENTARY DATA [file supp_gku673_nar-01291-met-g-2014-File008.pdf]

SUPPLEMENTARY MATERIAL

Broad-host-range vector system for synthetic biology and biotechnology in cyanobacteria

Arnaud Taton[1,*], Federico Unglaub[1], Nicole E. Wright[1], Wei Yue Zeng[1], Javier Paz-Yepez[2,3], Bianca Brahamsha[2], Brian Palenik[2], Todd C. Peterson[4,5], Farzad Haerizadeh[4,6], Susan S. Golden[1], and James W. Golden[1,*]

[1]Division of Biological Sciences, University of California San Diego, 9500 Gilman Dr., La Jolla, CA 92093, USA
[2]Scripps Institution of Oceanography, University of California San Diego, 9500 Gilman Dr., La Jolla, CA 92093, USA
[3]Institut de Biologie de l'Ecole Normale Supérieure, CNRS, UMR 8197, 46 rue d'Ulm, 75230 Paris, France
[4]Synthetic Biology Division, Life Technologies Corporation, 5791 Van Allen Way, Carlsbad, CA 92008, USA
[5]Present address: Synthetic Genomics, Inc. and Synthetic Genomics Vaccines, Inc, 11149 N Torrey Pines Rd., La Jolla, CA 92037, USA
[6]Present address: Intrexon Corporation, 6620 Mesa Ridge Rd, San Diego, CA 92121, USA

SUPPLEMENTARY METHODS

Method S1. ***In silico*** **construction of shuttle plasmids using the CYANO-VECTOR assembly portal.** Donor plasmid sequences and annotations are stored into BioBIKE, a Web-based, programmable, integrated biological knowledge base (1). BioBIKE also bears the codes written to assemble devices into shuttle plasmids, to display sequences and maps, as well as to write GenBank files and protocols. In cases where the resulting plasmid is to be displayed as a DNA sequence, BioBIKE uses its integrated sequence viewer. For GenBank files, BioBIKE writes text files that are directly viewable in a web browser. GenBank files are written so that color codes, associated to the different parts (or features), are retained in the program ApE (A plasmid Editor). ApE is a multi-platform plasmid editor, commonly used and freely available at http://biologylabs.utah.edu/jorgensen/wayned/ape/. Maps are produced as XML (Extensible Markup Language) files conforming to CGView instructions. The CGView application (2), under control of BioBIKE code, then creates SVG (Scalable Vector Graphics) files, readily viewable in a web browser. Protocols for the *in vitro* construction of shuttle plasmids from selected devices are written as Microsoft Office XML (Excel) files. The BioBIKE functions were made accessible outside of the BioBIKE environment through the CYANO-VECTOR assembly portal using the Web API (or Web service) functionalities of BioBIKE. These Web APIs allow an interface (in this case: a JavaScript enhanced html form) to communicate arguments (donor plasmids, custom sequences, or actions such as display map, display sequence, display GenBank file, or write a protocol) to BioBIKE. BioBIKE then processes the arguments to construct the desired plasmid *in silico* and writes the files required for the plasmid to be displayed, exported, or constructed *in vitro*.

Method S2. ***In vitro*** **construction of shuttle plasmids.** Detailed protocols for the construction of new shuttle plasmids can be generated from the CYANO-VECTOR assembly portal as a Microsoft Office XML (Excel) document. This document provides the list of donor plasmids to be used, and the mixture composition for the restriction digests and the assembly reaction. DNA concentrations of digested donor plasmids and any required PCR products are set such that there is an equimolar ratio of DNA modules and PCR products at a final concentration of about 8 ng/µl in the reaction. A worksheet entitled "Pooled digest and assembly" describes the mixture compositions of the restriction digests and the assembly reaction if the donor plasmids can be pooled dependent on the use of the same restriction enzyme. The worksheet entitled "Digest" and the worksheet entitled "Assembly" describe the mixture compositions of the restriction digests and the assembly reaction, respectively, if the donor plasmids are to be digested separately. The cells with green font, in particular those corresponding to DNA concentrations, must be edited according to the specific experiment.

SUPPLEMENTARY TABLES

Table S1. Strains and conjugal plasmids

Plasmid or strain	Relevant characteristic	Source
**Conjugal and helper plasmids**		
pRL443	Conjugal plasmid, Km^s derivative of RP4, Ap^r Tc^r	(3)
pRK2013	Conjugal plasmid, derivative of RK2, Km^r	(4,5)
pRL623	Helper plasmid carrying Mob_{ColK} and methylase genes M.AvaI, M.Eco47II, M.EcoT22I, Cm^r	(3)
pRL1045	Helper plasmid carrying Mob_{ColK} and methylase genes M.AvaI, M.Eco47II, Km^r	(3)
**Cyanobacterial strains (abbreviation)**		
Anabaena sp. PCC7120 (*A*. PCC7120)	Wild type	Laboratory Collection
Leptolyngbya sp. BL0902 (*L*. BL0902)	Wild type	(6)
Nostoc punctiforme ATCC29133 (*N*. ATCC29133)	Wild type	Michael L. Summers
Synechococcus elongatus PCC7942 (*S*. PCC7942)	Wild type	Laboratory Collection
Synechococcus sp. CC9311 (*S*. CC9311)	Wild type	(7)
Synechococcus sp. CC9605 (*S*. CC9605)	Wild type	(8)
Synechocystis sp. PCC6803 (*S*. PCC6803)	Wild type	Laboratory Collection
Synechocystis sp. WHSyn (*S*. WHSyn)	Wild type	Brian Palenik
***E. coli* strains**		
E. coli DH5α	Cloning host	Gibco BRL
E. coli DH10B	Cloning host	Gibco BRL
E. coli HB101	Cloning host	Gibco BRL
E. coli ED8654	Cloning host	(9)
E. coli TOP10	Cloning host	Invitrogen
E. coli ccdB survival	Cloning host	Invitrogen
E. coli AM0186	ED8654 harboring pRL443, Ap^r Tc^r	(3)
E. coli AM1219	HB101 harboring pRL1045, Km^r	(3)
E. coli AM1358	DH10B harboring pRL623, Cm^r	(10)
E. coli AM1359	DH10B harboring pRL623 and pRL443, Cm^r Ap^r Tc^r	(11)
E. coli AM4415	DH10B harboring pRK2013, Km^r	(6)

Table S2. Summary of devices organized according to the GC-adaptors

Device category	# Devices[a]	R.E.[b]	GC-adaptors	Adaptor sequences[c]
**Cyanobacterial replicons** (PDU1S, PDU1M, PDU1L, PDU1SZ, PDU1LZ, PFDA, PFDAZ, PDC1, PDC1Z, PANS)	11, 3	*Zra*I	G5C5-*Xba*I	5'-**GACGTC**GGGGGCCCCCGGGGGGATtctaga-3'
			*Sac*I-C3G3	5'-**GACGTC**GGGCCCGGGCCCGGGGATgagctc-3'
***E. coli* origin for knockout plasmids** (ORI-BOM-KO, ORI-BOM-KO-SACB)	2, 2			
***E. coli* origin to be assembled with a cyanobacterial replicon** (ORI-PMB1, ORI-PBAV1K)	2, 2	*Eco*RV	C3G3-*Mfe*I	5'-**GATATC**CCCGGGCCCGGGCCCGATcaattg-3'
			*Nhe*I-GC	5'-**GATATC**CGCGCGCGCGCGCGCGATgctagc-3'
**Broad-host-range replicon** (RSF1010, RSF1010Y25F, RSF1010Y25FK14*)	3, 3	*Zra*I	G5C5-*Xba*I	5'-**GACGTC**GGGGGCCCCCGGGGGGATtctaga-3'
			*Nhe*I-GC	5'-**GATATC**CGCGCGCGCGCGCGCGATgctagc-3'
**Neutral sites** (S7942NS1, S7942NS2, S7942NS3, S7942NS1-RK2BOM, S7942NS2-RK2BOM, S7942NS3-RK2BOM, S7942NS1-TC, S7942NS2-TC, A7120NS1-SACB, A7120NS2-SACB)	10,8			
**Antibiotic resistance markers** (AACC1, AADA, APHA1, BLA, BLA_A7120, BLA_S7942, CAT_A7120, CAT_S7942, ERM, HYGRO_A7120, HYGRO_S7942, NAT_A7120, NAT_S7942, NPTII, NPTII_A7120, PURO_A7120, PURO_S7942)	17, 17	*Eco*RV	GC-*Nhe*I	5'-**GATATC**GCGCGCGCGCGCGCGGATgctagc-3'
			*Age*I-C2G	5'-**GATATC**CGGCGGCGGCGGCGGGATaccggt-3'
**Cloning cassettes** (CCDB-SWAI)	1, 1	*Eco*RV	C2G-*Age*I	5'-**GATATC**CCGCCGCCGCCGCCGGATaccggt-3'
**Expression cassette** (PNI-CATCCDB-PMEI, CPTRC-GATEWAY**)**	2, 0		*Xba*I-G5C5	5'-**GATATC**CCCCCGGGGGCCCCCGATtctaga-3'
**Reporter cassette** (AHDI-YEMGFP, CATCCDB-AHDI-YEMGFP, CCDB-SWAI-ILOV-S7942, ZRAI-RIBOJ-YFP, CCDB-ZRAI-RIBOJ-YFP)	5, 2			
**Promoter-reporter module** (PCONII-GFPMUT2, PCONII-YEMGFP, PCONII-YFP, PCONII-ECFP, PPSBAI-GFPMUT2, PPSBAI-YFP, PPHOA-GFPMUT2, PPHOA-YFP, PISIA-GFPMUT2, PISIA-YFP, PCONII-SWAI-RIBOJ-YFP, PCONII-SWAI-RIBOJ-ECFP, PCONII-SSPI-RIBOJ-YFP, PCONII-SSPI-RIBOJ-ECFP, PCONII-SWAI-YFP, PCONII-SWAI-ECFP, PCONII-ILOV_S7942)	17, 15			

[a] The numbers of devices are presented as follows: # constructed, # tested.
[b] Restriction enzyme to be used for releasing the device from the donor plasmid.
[c] The *Eco*RV or *Zra*I restriction site to release the device from the donor plasmid is written in bold and uppercase, the core of the adaptor sequence is written in uppercase and an additional restriction site is written in lowercase.

Table S3. Assembled shuttle plasmids to evaluate antibiotic resistance markers and variants of the RSF1010 replicon in 4 different strains of cyanobacteria

Strain	Plasmid	Plasmid components	P.A.E.	*S.* PCC7942	*A.* PCC7120	*L.* BL0902	*S.* PCC6803
AM4913	pCV0025	NS1TC***aadA***P*con*II-yfp	12/12	+			
AM4915	pCV0027	NS1TC***aphI***P*con*II-yfp	7/7	+			
AM4914	pCV0026	NS1TC***aacC1***P*con*II-yfp	5/6	+			
AM4918	pCV0030	NS1TC***bla_A7120***P*con*II-yfp	/	+			
AM4919	pCV0031	NS1TC***bla_S7942***P*con*II-yfp	5/7	(+)			
AM4916	pCV0028	NS1TC***cat_A7120***P*con*II-yfp	6/6	+			
AM4917	pCV0029	NS1TC***cat_S7942***P*con*II-yfp	6/6	+			
AM4920	pCV0032	NS1TC***nat1_A7120***P*con*II-yfp	6/6	+			
AM4921	pCV0033	NS1TC***nat1_S7942***P*con*II-yfp	6/6	+			
AM4889	pCV0001	**RSF1010Y25F*aadA***P*con*II-GFPmut2	/		+	+	+
AM4891	pCV0003	RSF1010Y25F***aphI***P*con*II-GFPmut2	/		+	+	+
AM4890	pCV0002	RSF1010Y25F***aacC1***P*con*II-GFPmut2	/		+	+	+
AM4894	pCV0006	RSF1010Y25F***bla_A7120***P*con*II-GFPmut2	/		(+)	(+)	(+)
AM4895	pCV0007	RSF1010Y25F***bla_S7942***P*con*II-GFPmut2	/		(+)	(+)	(+)
AM4892	pCV0004	RSF1010Y25F***cat_A7120***P*con*II-GFPmut2	3/3		+	+	+
AM4893	pCV0005	RSF1010Y25F***cat_S7942***P*con*II-GFPmut2	/		+	+	+
AM4896	pCV0008	RSF1010Y25F***nat1_A7120***P*con*II-GFPmut2	3/3		+	+	+
AM4897	pCV0009	RSF1010Y25F***nat1_S7942***P*con*II-GFPmut2	3/3		+	+	+
AM4996	pCV0085	**RSF1010WT***aadA*P*con*II-GFPmut2	1/1		+	+	+
AM4962	pCV0074	**RSF1010Y25FK14****aadA*P*con*II-GFPmut2	/		+	+	-

+ Successful transformation/conjugation; (+) Problematic segregation of the cargo strain; (P.A.E.) Plasmid assembly efficiency, see text for explanation. For testing in *S.* PCC7942 antibiotic resistance genes were cloned into the NS1TC vector; for all other strains, the antibiotic resistance genes were cloned into the RSF1010Y25F plasmid. Gray areas block off inapplicable host/vector combinations.

Table S4. Assembled shuttle plasmids used to evaluate promoters and reporter genes in 4 different strains of cyanobacteria

Strain	Plasmid	Plasmid components	P.A.E.	*S.* PCC7942	*A.* PCC7120	*L.* BL0902	*S.* PCC6803
AM4913	pCV0025	NS1TC*aadA*PconII-yfp	/	+			
AM4922	pCV0034	NS1TC*aadA*PconII-GFPmut2	3/6	++			
AM4923	pCV0035	NS1TC*aadA*PconII-yemGFP	5/5	+++			
AM4924	pCV0036	NS1TC*aadA*PconII-eCFP	6/6	-			
AM4927	pCV0039	NS1TC*aadA*PpsbAI-GFPmut2	4/4	+			
AM4932	pCV0044	NS1TC*aadA*PconII-riboJ-oRBS-YFP	8/8	+++			
AM4933	pCV0045	NS1TC*aadA*PconII-oRBS-YFP	/	+++			
AM4935	pCV0047	NS1TC*aadA*PconII-riboJ-oRBS-eCFP	6/6	++			
AM4936	pCV0048	NS1TC*aadA*PconII-oRBS-eCFP	8/8	++			
AM4903	pCV0015	RSF1010Y25F*aadA*PconII-GFPmut2	/		++	++	++
AM4899	pCV0011	RSF1010Y25F*aadA*PconII-yfp	/		-	-	-
AM4898	pCV0010	RSF1010Y25F*aadA*PconII-yemGFP	/		-	+++	++
AM4900	pCV0012	RSF1010Y25F*aadA*PconII-eCFP	/		-	-	-
AM4903	pCV0015	RSF1010Y25F*aadA*PpsbAI-GFPmut2	4/4		++	+	-
AM4908	pCV0020	RSF1010Y25F*aadA*PconII-riboJ-oRBS-YFP	8/8		+++	+++	+++
AM4909	pCV0021	RSF1010Y25F*aadA*PconII-oRBS-YFP	3/3		+++	+++	+++
AM4911	pCV0023	RSF1010Y25F*aadA*PconII-riboJ-oRBS-eCFP	8/8		++	++	++
AM4912	pCV0024	RSF1010Y25F*aadA*PconII-oRBS-eCFP	8/8		++	++	++
AM4925	pCV0037	NS1TC*aadA*PisiA-GFPmut2	4/4		*	*	*
AM4926	pCV0036	NS1TC*aadA*PphoA-GFPmut2	4/4		*	*	*
AM4901	pCV0013	RSF1010Y25F*PisiA-GFPmut2	4/4		*	*	*
AM4902	pCV0014	RSF1010Y25F *aadA*PphoA-GFPmut2	4/4		*	*	*
AM4995	pCV0084	NS1RK2BOM*aadA*Empty	4/4		-	-	-
AM4967	pCV0079	RSF1010Y25F *aadA*Empty	4/4		-	-	-

Fluorescence levels: -, undetectable; +, dim; ++, bright, +++, very bright; * regulated promoters, data shown in Figure 6; (P.A.E.) Plasmid assembly efficiency, see text for explanation. Gray areas block off inapplicable host/vector combinations.

SUPPLEMENTARY FIGURES

Figure S1. CYANO-VECTOR assembly web portal showing (A) the portal home page, (B) an example of the map of a newly assembled plasmid, (C) its sequence, and (D) the protocol template for the assembly reaction. Any number in green can or must be adjusted according to the experiment, for example, DNA concentrations must be entered. If DNA concentrations are too high, the dilution factor (D.F.) can be adjusted. The concentration of devices in the assembly reaction should be about 8 ng/µl. This can be adjusted by changing the "amount of fragment per kb". The default reaction volume of 10 µl can be changed; for example, 5-µl volumes work well. Finally, equimolar ratios of DNA devices and PCR products (1,1,1,1) work well in most cases but can also be adjusted.

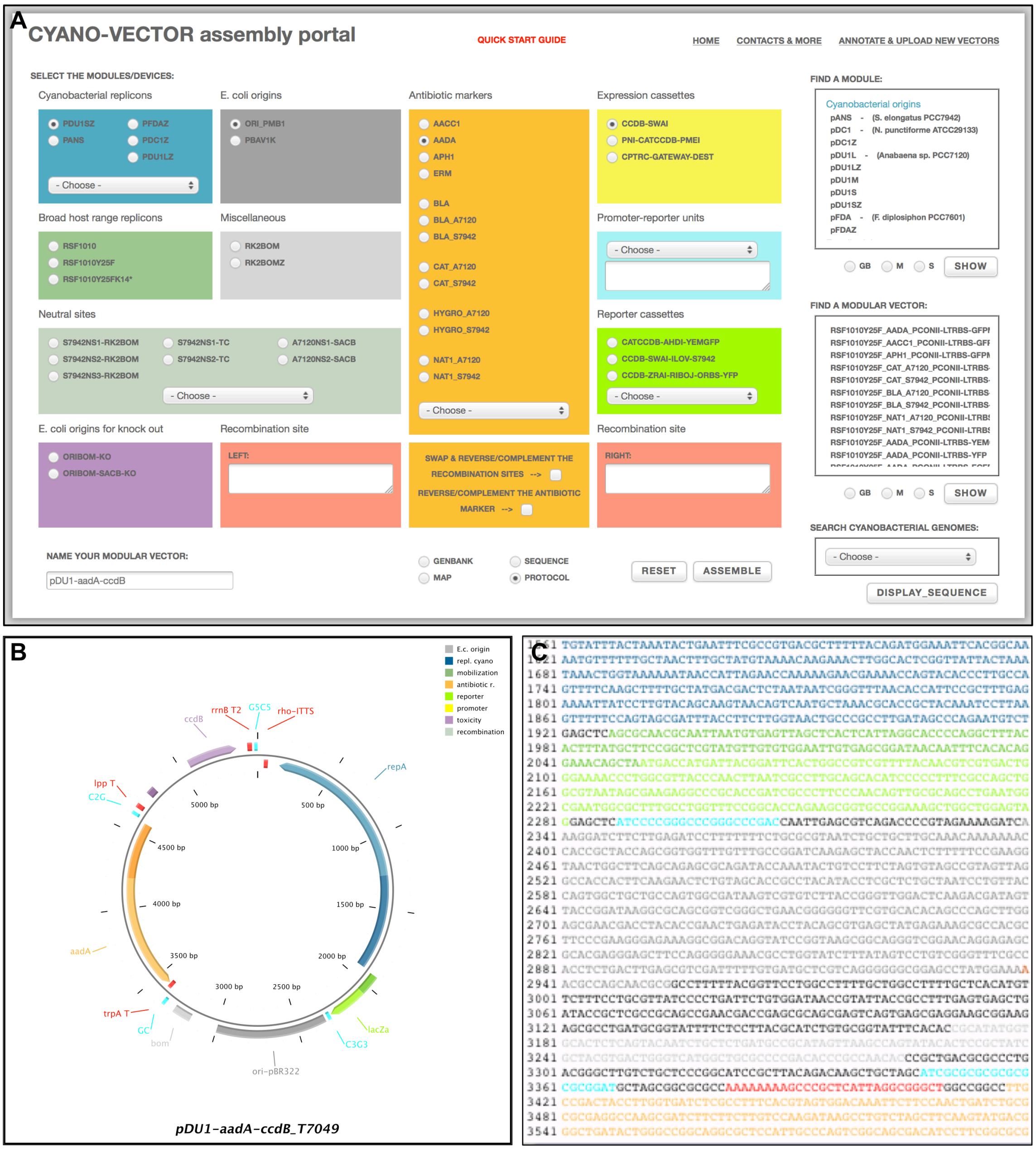

D

**Calculation**	Device (nt)	Vector (nt)	Ratios	Vector (ng/ul)	Device (ng)	Vector (ng)	Vector (ul)	D.F.
pCVD055	2329	5021	1	100	34.94	75.32	0.75	1
pCVD026	1080	3772	1	100	16.20	56.58	0.57	1
pCVD002	1276	3623	1	100	19.14	54.35	0.54	1
pCVD015	893	3585	1	100	13.40	53.78	0.54	1

Amount of fragment per Kb: 15 ng — 8 ng/ul of part DNA in assembly reaction

Volume of the assembly reaction: 10 ul

**Invitrogen - Seamless**	Final		Stocks		Volume	
Grand total/Water	10	ul			4.60	ul
pCVD055	75.32	ng	100	ng/ul	0.75	ul
pCVD026	56.58	ng	100	ng/ul	0.57	ul
pCVD002	54.35	ng	100	ng/ul	0.54	ul
pCVD015	53.78	ng	100	ng/ul	0.54	ul
Buffer	1	x	5	x	2	ul
Enzyme	1	x	10	x	1	ul
				Grand Total:	10.00	ul

Pooled digests and assembly | Digests | Assembly

Figure S2. Evaluation of conjugal efficiencies of 3 variants of the RSF1010 replicon (WT, mobAY25F and mobAY25F/mobCK14*) in *Anabaena* sp. PCC7120, *Leptolyngbya* sp. BL0902 and *Synechocystis* sp. PCC6803. Biparental matings were carried out with the same amount of cyanobacteria and *E. coli* cells for each variant (pCV0001, pCV0084, pCV0079; Table S3), and then serial dilutions of the mating mixtures were spotted on plates. The experiment was carried out with 3 biological replicates for each variants of the RSF1010 replicon (3 cultures of *E. coli* grown from 3 independent colonies) and for each mating; furthermore 2 technical replicates were carried out. The results presented were highly reproducible. Note that most of what appears as small colonies at the dilution factor 1:5 for *Synechocystis* sp. PCC6803 and the RSF1010 replicon variant *mobA*Y25F are not real.

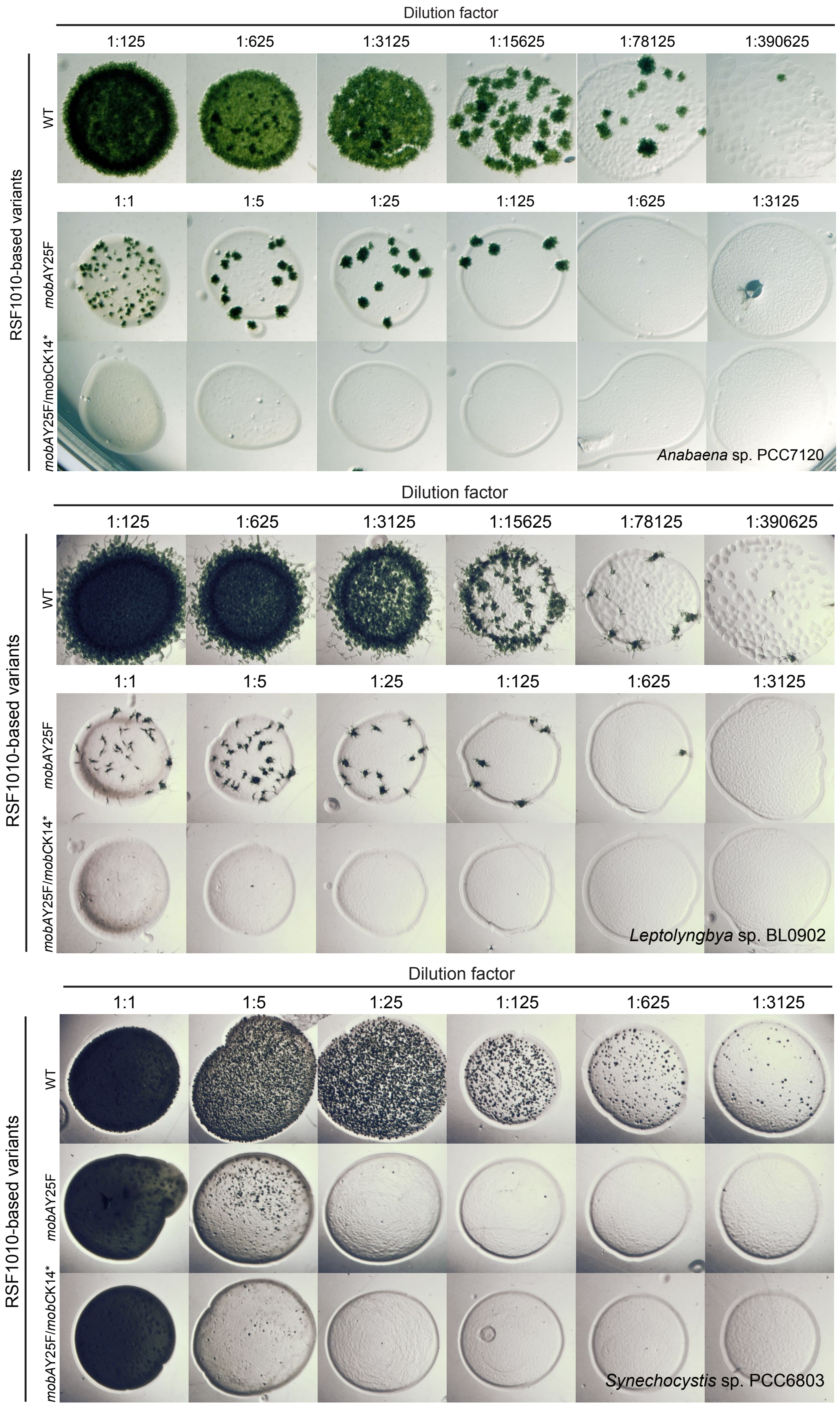
Dilution factor
1:125
1:625
1:3125
1:15625
1:78125
1:390625
1:1
1:5
1:25
1:125
1:625
1:3125
RSF1010-based variants
WT
mobAY25F
mobAY25F/mobCK14*
Anabaena sp. PCC7120
Dilution factor
1:125
1:625
1:3125
1:15625
1:78125
1:390625
1:1
1:5
1:25
1:125
1:625
1:3125
RSF1010-based variants
WT
mobAY25F
mobAY25F/mobCK14*
Leptolyngbya sp. BL0902
Dilution factor
1:1
1:5
1:25
1:125
1:625
1:3125
RSF1010-based variants
WT
mobAY25F
mobAY25F/mobCK14*
Synechocystis sp. PCC6803

Figure S3. PCR assays showing the stability, presence or absence, of plasmids based on the RSF1010(*mobA*Y25F) replicon in several strains of cyanobacteria. (A) Three pairs of primers (1 - mobC_d412R, 5’-CGCTAAACCCCACACCAAACCC-3’ and repA_146R, 5’-TGCAGGGCCAGCATGGATTTAC-3’; 2 - repB_247F, 5’-GACCTGGATGACATGAAAGCCG-3’ and repC_718R, 5’-CCTCTGACGGCCAGACATAGC-3’; 3 - mobC_216F 5’-GTACCTTGAACGCGACCACGAC-3’ and repC_577F, 5’-CGCATCAGCATGGACGAGGTG-3’) were used to amplify overlapping PCR products covering the entire plasmid pCV0021 (Table S4) isolated from *E. coli*-free strains of *Anabaena* sp. PCC7120 (A7120), *Leptolyngbya* sp. BL0902 (L0902), *Synechocystis* sp. PCC6803 (S6803) and *Synechocystis* sp. WHSyn (WHSyn). Transconjugant strains were grown for 4 months (L0902 and WHSyn) or 8 months (A7120 and S6803) in liquid media supplemented with 2 µg/ml of both spectinomycin and streptomycin, and were transferred in fresh medium every 2 to 4 weeks. PCR products were obtained with each pair of primers from all four transconjugant strains (upper panels) and the plasmid control (P.c., lower panels), but not from WT strains (lower panels) or a blank control (upper panels). (B) One pair of primers (GFP-FW 5’-AGTAAAGGAGAAGAACTTTTCA-3’ and GFP-RV, 5’-ccgcgtttccagactttacTTATTTGTATAGTTCATCCATGC-3’) was used to detect the presence of pCV0003 (Table S3) in *E. coli*-free strains of *Synechococcus* sp. CC9311 and CC9605 grown over a period of 4 months after the first, third and eighth transfer of kanamycin resistant strains in fresh liquid medium supplemented with 100 µg/ml of kanamycin. After kanamycin resistant colonies were grown for 2 weeks in liquid medium, PCR products were obtained for 17 transconjugant lines whereas as many lines appeared to have acquired resistance to kanamycin without carrying the plasmid. PCR products were obtained from the plasmid control (P.c.), but not from WT strains (WT) or the blank control. After 6 weeks, PCRs carried out on 8 transconjugant lines of *Synechococcus* sp. CC9311 and CC9605 still showed the presence of the plasmid whereas the plasmid could not be detected anymore after 18 weeks. The PCRs were carried out directly on 1 µl of low-density liquid culture (boiled prior to PCRs), ~10 ng of plasmid DNA for the plasmid controls, or 1 µl of water for the blanks. See text for discussion.

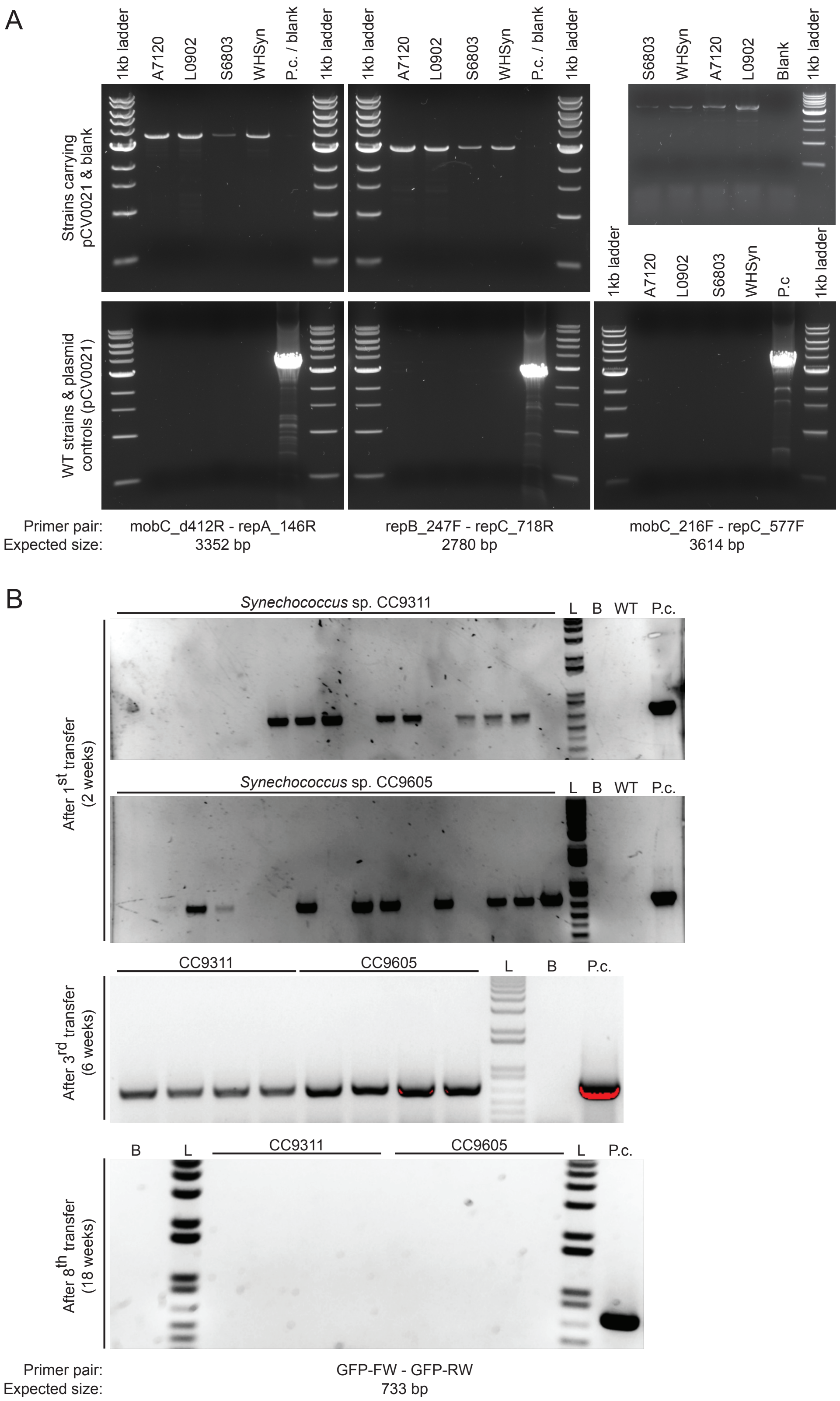
A
1kb ladder
A7120
L0902
S6803
WHSyn
P.c. / blank
Blank
P.c
Strains carrying pCV0021 & blank
WT strains & plasmid controls (pCV0021)
Primer pair:
Expected size:
mobC_d412R - repA_146R
3352 bp
repB_247F - repC_718R
2780 bp
mobC_216F - repC_577F
3614 bp
B
Synechococcus sp. CC9311
Synechococcus sp. CC9605
L
B
WT
P.c.
After 1st transfer (2 weeks)
CC9311
CC9605
After 3rd transfer (6 weeks)
After 8th transfer (18 weeks)
GFP-FW - GFP-RW
733 bp

SUPPLEMENTARY REFERENCES

1. Elhai, J., Taton, A., Massar, J.P., Myers, J.K., Travers, M., Casey, J., Slupesky, M. and Shrager, J. (2009) BioBIKE: a Web-based, programmable, integrated biological knowledge base. *Nucleic Acids Res*, **37**, W28-32.

2. Stothard, P. and Wishart, D.S. (2005) Circular genome visualization and exploration using CGView. *Bioinformatics*, **21**, 537-539.

3. Elhai, J., Vepritskiy, A., Muro-Pastor, A.M., Flores, E. and Wolk, C.P. (1997) Reduction of conjugal transfer efficiency by three restriction activities of *Anabaena* sp. strain PCC 7120. *J Bacteriol*, **179**, 1998-2005.

4. Cohen, M.F., Wallis, J.G., Campbell, E.L. and Meeks, J.C. (1994) Transposon mutagenesis of *Nostoc* sp. strain ATCC 29133, a filamentous cyanobacterium with multiple cellular differentiation alternatives. *Microbiology*, **140**, 3233-3240.

5. Wu, X., Lee, D.W., Mella, R.A. and Golden, J.W. (2007) The *Anabaena* sp. strain PCC 7120 asr1734 gene encodes a negative regulator of heterocyst development. *Mol Microbiol*, **64**, 782-794.

6. Taton, A., Lis, E., Adin, D.M., Dong, G., Cookson, S., Kay, S.A., Golden, S.S. and Golden, J.W. (2012) Gene transfer in *Leptolyngbya* sp. strain BL0902, a cyanobacterium suitable for production of biomass and bioproducts. *PLoS ONE*, **7**, e30901.

7. Palenik, B., Ren, Q., Dupont, C.L., Myers, G.S., Heidelberg, J.F., Badger, J.H., Madupu, R., Nelson, W.C., Brinkac, L.M., Dodson, R.J. *et al.* (2006) Genome sequence of *Synechococcus* CC9311: Insights into adaptation to a coastal environment. *Proc Natl Acad Sci U S A*, **103**, 13555-13559.

8. Toledo, G. and Palenik, B. (2003) A *Synechococcus* serotype is found preferentially in surface marine waters. *Limnology and oceanography*.

9. Borck, K., Beggs, J.D., Brammar, W.J., Hopkins, A.S. and Murray, N.E. (1976) The construction in vitro of transducing derivatives of phage lambda. *Mol Gen Genet*, **146**, 199-207.

10. Liu, D. and Golden, J.W. (2002) *hetL* overexpression stimulates heterocyst formation in *Anabaena* sp. strain PCC 7120. *J Bacteriol*, **184**, 6873-6881.

11. Yoon, H.S. and Golden, J.W. (1998) Heterocyst pattern formation controlled by a diffusible peptide. *Science*, **282**, 935-938.
